# COMMUNICATION AND WORKFLOW ON A TYPICAL DAY IN NH VERSUS A DAY WITH A CHANGE IN RESIDENT CONDITION

**DOI:** 10.1093/geroni/igac059.2564

**Published:** 2022-12-20

**Authors:** Alyce Ashcraft, Donna Owen, John Culberson, Huaxin Song

**Affiliations:** Texas Tech University Health Sciences Center, Lubbock, Texas, United States; Texas Tech University Health Sciences Center, Lubbock, Texas, United States; Texas Tech University Health Sciences Center, Lubbock, Texas, United States; Texas Tech University Health Sciences Center, Lubbock, Texas, United States

## Abstract

A major reason technologic innovations for nursing home (NH) communication have not worked is because testing of technology fails to consider workflow of the environment. The aim of this observational study was to examine communication and workflow surrounding assessment of NH residents on a dementia care unit with suspected UTI to establish baseline data prior to introduction of technology to enhance NH staff communication. The flow of communication between CNAs, LVNs, and PCPs was recorded using a structured observation on an online survey platform. For a 3-month period, 3 days a week, an observation period from 7am to 11am was chosen to reflect one peak staff interaction time. Field notes describing the environment and what was being talked about during communication events were analyzed using content analysis. A total of 185 communication events were recorded by 3 trained observers, yielding 22 assessments for change in condition (pain, UTI, falls). The LVN was the center of 44% of communication. After the LVN assessed the resident, 43% of the time no further action was taken by the LVN; 17% of the time the PCP was texted about a change in condition. Types of change in condition was limited. Workflow surrounding LVN collection of information on days related to change in condition reflected focused communication among staff occurring away from resident rooms and nurses’ station. The NH is a stable environment for provision of nursing care, thus suggesting communication technology can be accepted by staff and added into the care routine.

